# Evolution of body morphology and beak shape revealed by a morphometric analysis of 14 Paridae species

**DOI:** 10.1186/s12983-016-0162-0

**Published:** 2016-06-29

**Authors:** Shimiao Shao, Qing Quan, Tianlong Cai, Gang Song, Yanhua Qu, Fumin Lei

**Affiliations:** Key Laboratory of Zoological Systematics and Evolution, Institute of Zoology, Chinese Academy of Sciences, Beijing, 100101 China; University of Chinese Academy of Sciences, Beijing, 100049 China; Guangdong Entomological Institute, Chinese Academy of Sciences, Guangdong, 510260 China

**Keywords:** Morphology, Paridae, Geometric morphometrics, Phylogenetic relationship, Altitude, Distribution overlap

## Abstract

**Background:**

Morphological characters of birds reflect their adaptive evolution and ecological requirements and are also relevant to phylogenetic relationships within a group of related species. The tits (Paridae) are known to be outwardly homogeneous in shape, with one aberrant member, the Ground Tit (*Pseudopodoces humilis*), which is quite different from its relatives in both body morphology and beak shape. We combined traditional measurements and geometric morphometrics to quantify the variation in body morphology and beak shape of 14 Paridae species distributed in China. Based on these results, we sought to assess the contribution of phylogeny, altitude and species interactions to the evolution of morphological traits.

**Results:**

The basic features for discriminating among the 14 species studied here were overall body size, the ratio of body and tail length to culmen and tarsus length, and beak shape (long/slender/pointy vs. short/robust/blunt). These dimensions clearly separate *Ps. humilis* and *Melanochlora sultanea* from the other species in shape space. Body length and PC3 of beak shape (round outline vs. straight outline) show significant phylogenetic signals. Across 14 species, altitude is related to tarsus, culmen length and PC1 of beak shape. Within *Parus major*, altitude is related to body weight, body length, culmen length and PC1 of body morphology. Morphological distances and geographic distances among species are positively correlated.

**Conclusions:**

The body morphology of Paridae species shows extensive evolutionary changes, while their beak has mainly evolved along the long/slender/pointy vs. short/robust/blunt dimension. Only body length and beak curvature show a phylogenetic signal. Altitude correlates with multiple traits both across and within species, suggesting that altitude is an important factor in promoting morphological divergence. The deviant appearance of *Ps. humilis* corresponds to its foraging and feeding adaptations to high-altitude steppe habitats. Our results also show a higher level of morphological divergence with greater difference in distribution ranges among the Paridae species involved in this study.

**Electronic supplementary material:**

The online version of this article (doi:10.1186/s12983-016-0162-0) contains supplementary material, which is available to authorized users.

## Background

Diversity among a group of related organisms usually shows a pattern of descent with modification, with both phylogenetic history and natural selection influencing a species’ form [1, 2]. A bird’s body size and shape are related to its migratory habits, flight mode, habitat use, and foraging behaviour, as well as the effects of sexual selection [3–5]. Bird beaks are considered relevant to foraging modes, food types, parental care and song structure [6–8]. Morphological divergence, which enables related organisms to avoid competition and exploit available niches, plays an important role in adaptive radiation [9, 10]. Previous studies have shown that rapid diversification in adaptive radiations usually involve accelerated rates of morphological evolution [11, 12]. Therefore, morphological characters are frequently used to investigate adaptation radiation, such as beak shape adapting to different food sources in Darwin’s finches [13], extremely diverse beak morphology of the Hawaiian honeycreepers [14], and the speciation bursts of Malagasy vangas related to morphological diversification [10].

The avian Family Paridae, which includes c. 55 species that are distributed across the northern hemisphere, tropical Africa and Indonesia and are found from sea level to 5500 m [15, 16], is a well-investigated example of adaptive radiation. The hotspot of Paridae species richness is found in China, which is also considered as the centre of origin of this group and plays a role as a “cradle of diversity” [17]. Previous studies have proposed that the diversification of Paridae is primarily driven by vicariance related to orogenesis in the Himalayas and Mountains of Southwest China concurrent with the uplift of the Qinghai-Tibet Plateau (QTP) [17, 18]. Paridae species have diverse plumage within their large family but outwardly appear rather uniform in shape, except for the Sultan Tit (*Melanochlora sultanea*), the Yellow-browed Tit (*Sylviparus modestus*) and the Ground Tit (*Pseudopodoces humilis*) [16, 19]. *Pseudopodoces humilis* is endemic to the QTP and dwells exclusively on the treeless steppes, which has been identified as an extreme example in the evolution of Paridae species [16, 20]. It had long been misclassified as a small species of ground jay due to its strikingly aberrant appearance, while recent studies have revealed that this species is closely related to *Parus major* and *P. monticolus,* with a divergent time of approximately 7.7 - 9.9 million years [21–23]. The extremely deviant morphology of *Ps. humilis* has received extensive attention, and qualitative descriptions of its morphological differences from other parids have been documented [21]. In addition, univariate analyses of linear characters, such as body size and beak length, have been performed on a few Paridae species to investigate interspecific interactions, foraging behaviour and niche shifts [24, 25]. However, few studies have quantified morphological variation across the main clades and investigated potential factors that could shape the patterns of diversity in this family.

In this study, we first aimed to quantify the variation in body morphology and beak shape of 14 Paridae species distributed in China. We applied traditional morphometric methods to analyse body morphology. For beak morphology, we first measured culmen length, but linear distances such as beak length, width and depth are insufficient to fully describe beak shape [26–28]. Geometric morphometric methods can capture the explicit geometry of a morphological structure by examining associations among an entire set of landmarks and has the additional advantages of improved statistical power and fewer a priori assumptions about what should be measured [27, 29, 30]. These methods have increasingly become a powerful and useful tool to study morphological variation and adaptive radiation, especially when investigating features such as bird beaks [30] and skulls [8, 31, 32]. Therefore, we applied the improved inferential resolution of geometric morphometric methods, through which we attempted to present visible and interpretable variations in beak shape.

We then examined the factors that could potentially affect morphological variation. Firstly, we tested for phylogenetic signal in each morphological trait to evaluate whether species that descended from a recent common ancestor closely resembled each other. Secondly, we assessed the correlation between morphology and altitudinal distribution. Altitude is associated with predictable changes in temperature, precipitation and habitat type, which result in different selective pressures and consequently influence the fauna that are present along altitudinal gradients [33, 34]. Adaptation to different altitudes may result in the divergence of phenotypes between populations and eventually contribute to speciation. To test this hypothesis, we looked for morphological variation along the altitudinal gradient both among Paridae species and within *P. major*, which occurs from 400 m to as high as 4400 m above sea level. Lastly, we examined the correlation between character divergence and distribution overlap to test the influence of species interactions on morphology. Ecological differences are essential to coexistence, and closely related, sympatric species need to differ in at least one niche dimension of habitat selection, prey size and feeding method to avoid competition [35–37]. Ecological variation along these dimensions is associated with predictable morphological changes [36]. Thus, we expected greater character divergence among Paridae species with greater overlap in their distribution range.

## Methods

### Data collection

We measured 376 museum specimens belonging to 14 species (see Additional file 1: Table S1 for a species list) from the National Zoological Museum of China, Institute of Zoology, Chinese Academy of Sciences. Data for body weight and body length were retrieved from the original records taken during field collection. Wing length, tail length, tarsus length and culmen length were measured to the nearest 0.1 mm with a digital caliper (see Additional file 1: Table S2 for detailed methods of measurement and Additional file 2 for a complete description of the data). For geometric morphometric analyses, the beak profiles of 306 specimens were captured using an image collecting system consisting of a Keyence VHX-1000C microscope and a Nikon Ni-E camera. Only adult males were included. All individuals of *P. major* were from *minor* section [19].

### Body morphology

Before statistical analyses, all measurements were log-transformed to normalize their distributions [4]. We performed one-way ANOVAs to compare each character among the species. In addition, we conducted a canonical variate analysis (CVA) on the data of all individuals to extract axes that best discriminated among the groups and to generate a matrix of pairwise Mahalanobis distances [38].

A principal component analysis (PCA) was applied to species mean values of each character to reduce the number of variables and visualize the variation. Before the PCA, all data were standardized to a mean of zero and a variance of one to minimize the effect of different initial units. Body size is important for evolutionary and ecological studies [39]; therefore, the data were not size-corrected before the PCA to preserve the information of body size.

### Beak shape variation

**Fig. 1:**
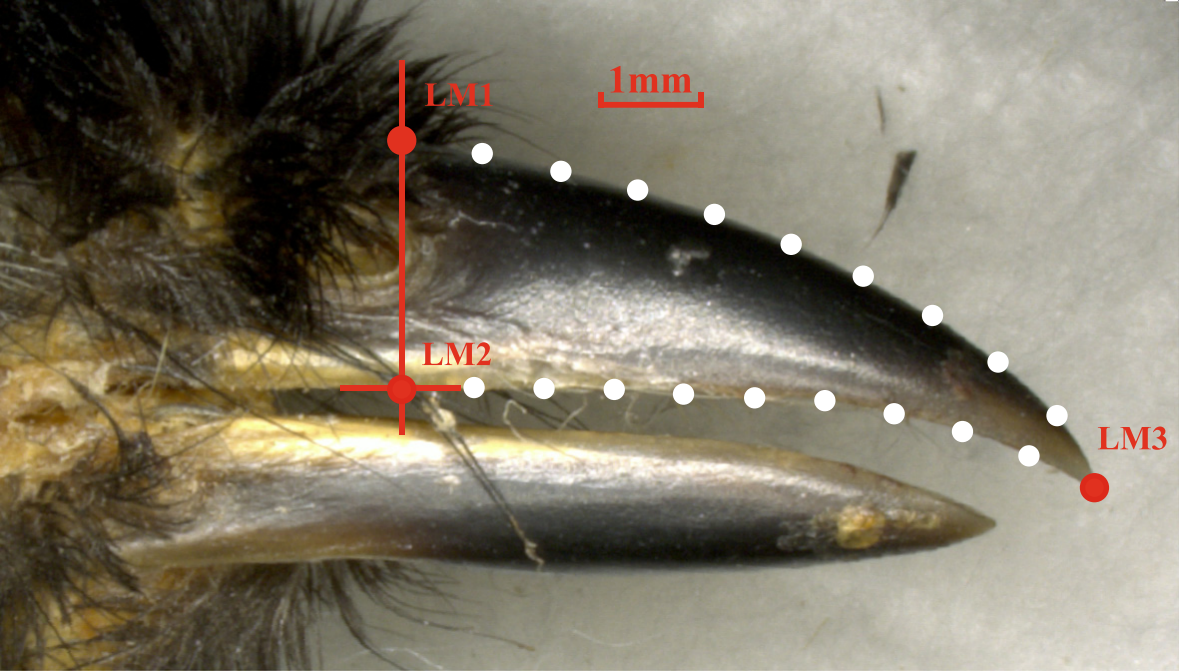
Landmarks and semi-landmarks used for the geometric morphometric analysis. A line perpendicular to the suture was drawn across the rostral edge of the nares. Two landmarks were placed where this line intersects the outline of the upper mandible, whereas the third was placed at the tip of the beak. Nine semi-landmarks were placed equidistantly between LM1 and LM3, and the other nine between LM2 and LM3

To characterize the shape of the beaks, we analysed the profile of part of the upper mandible from the nares to the tip because the whole lateral view of the beak is difficult to capture due to the varied rictal bristles and feather coverage. TpsDig [40] was used to place 3 landmarks and 18 semi-landmarks [29] along the outline of the beak (see Fig. 1). The semi-landmarks, which were important for quantifying the shape of the beak, which lacks clear homologous points, were placed equidistantly. We used tpsRelw [41] to slide the semi-landmarks and then output the aligned specimens (see Additional file 3 for the txt file of aligned specimens), which was later analysed for shape variation using MorphoJ [42]. The CVA was performed on all configurations to extract the axes with the greatest interspecific differences and generate a matrix of pairwise Mahalanobis distances [38]. A Procrustes ANOVA [43] was conducted as extra effects. The PCA was performed on species mean shapes to visualize the shape changes that accounted for most of the variation.

Geometric morphometrics use superimposition methods to eliminate non-shape variation in the configurations of the landmarks by overlaying them according to some optimization criterion [26]. In this study, we applied Procrustes generalized least squares. This method first translates the centroid of each configuration to the origin, then divides the configurations by centroid size to scale them to a common unit of size and finally optimally rotates each configuration to minimize the squared differences between corresponding landmarks [44, 45]. This procedure eliminates the effects of position, scale and orientation so that the Procrustes coordinates of landmarks of a configuration can represent size-free shape, while the centroid size represents overall beak size.

### Phylogenetic and comparative approach

To serve as the backbone for a comparative study, a phylogenetic tree needs to show a branching pattern and estimates of the branch lengths, which are assumed to be proportional to the expected variance in the amount of evolutionary change along each branch under a Brownian-motion model [46]. The phylogeny of Paridae has been extensively investigated in recent decades, and some taxonomic revisions have been proposed [15, 47, 48]. In this study, we followed the complete phylogeny of Paridae presented by Johansson et al. [47] and constrained the topology of the 14 species involved. The branch lengths were then calculated using two nuclear genes (*Myoglobin* and *Ornithine Decarboxylase*) and two mitochondrial genes (*NADH dehydrogenase subunit 2* and *Cytochrome b*). We used MrBayes 3.2 [49] for the phylogenetic construction with gene partitions, using best-fit models based on BIC model selection criteria (GTR + I + G, TIM + I + G, K80 + G and HKY + G for *Cytb*, *ND2*, *Myo*, and *ODC*, respectively). The sequences were downloaded from GenBank (see Additional file 1: Table S3 for accession numbers and references).

The reconstructed phylogenetic tree (see Additional file 4) was then projected into the tangent shape space of the PC scores computed from the mean beak shape of each terminal taxon to show the trajectories of specific lineages and clades. The ancestral states of internal nodes were reconstructed based on squared-change parsimony [50], which is identical to assuming Brownian motion.

Blomberg’s K [51] and Pagel’s λ [52] were used to assess the phylogenetic signal in morphological traits. For both indices, a value close to 0 indicates phylogenetic independence and a value of 1 indicates that traits are evolving according to Brownian motion in the given phylogeny. Values that are higher than 1 indicate that traits are more similar amongst species than would be expected due to a Brownian-motion model [51–53]. The residuals of regressions of log-transformed linear traits on log-transformed body weight values were used as size-corrected traits [35]. We tested for phylogenetic signal in log-transformed linear traits, size-corrected traits and PC values calculated from body morphology and beak shape.

### Correlation between morphology and altitude

We used regression analyses to investigate covariation between morphological traits and altitudinal distribution. Analyses were carried out at two hierarchical levels: 1) variation among the 14 species and 2) variation within *P. major*. For determining interspecific covariation, a regression of species mean values of each morphological trait on the mid-points of species’ altitudinal ranges was conducted. And phylogenetic generalized least square (PGLS) means were applied to correct for phylogenetic relationships [54]. The altitudinal ranges of each species were determined based on values reported in the literature [19, 55]. As for the intraspecific covariation, we used data of individuals within *P. major* because the large sample size of this species enabled the intraspecific analyses to be conducted. The altitudinal data of each *P. major* specimen was collected from the original records of the sampling sites. For specimens that lacked records of the sampling sites, altitudinal data were substituted with the mid-points of the altitudinal ranges of populations. For both interspecific and intraspecific analyses, each morphological character was separately correlated with altitude. We conducted each regression twice and then used the mean value of these replicates as the final result to minimize type I errors.

### Character divergence and distribution overlap

We conducted a partial Mantel test [56] (10,000 permutations) to look for possible relationships between character divergence and the degree of sympatry between species. The test was performed on the matrices of pairwise Mahalanobis distances of the morphological characters (both body morphology and beak shape) and the matrices of distributional distance, with the phylogenetic distances included as controlled variables. Both geographic distribution and altitudinal distribution were investigated.

As all specimens we measured were from China, their morphological variation may be insufficient to explain global patterns due to the considerable intraspecific differences in morphology between populations in China and those in other areas. Therefore, for each species, we used only the distribution range within China. We drew maps of the geographical distributions for all species on a Global Information System (GIS) (ArcMap 10.2) and calculated the degree overlap using the following equation: D_O_ = O_AB_ / (S_A_ + S_B_ – O_AB_), where S_A_ and S_B_ are the areas of the ranges of species A and species B, and O_AB_ is the area in which their ranges overlap. Furthermore, D_AB_ = 1 – D_O_ was used to define the geographical distance. Distribution data were extracted from BirdLife International and NatureServe [57]. The degree of altitudinal overlap between species was also calculated using this method.

We used PAST 2.17 [58] to perform the PCA, ANOVA, ANCOVA, CVA of body morphology and regression analysis; MorphoJ [42] for the geometric morphometric analysis of variation in beak shape; the package ape [59], geiger [60] and phytools [61] in R for phylogenetic signal test; the package nlme [62] in R for the PGLS analysis; and the package vegan [63] in R for the Mantel test. The phylogenetic signal test and the Mantel test were performed separately on the dataset including all 14 species and on the dataset without *Ps. humilis*.

## Results

### Variation in body morphology

One-way ANOVAs of the linear measurements show significant interspecific differences for all six characters (see Additional file 1: Table S4 for summary statistics and Additional file 1: Figure S1 for box plots). A CVA conducted on all individuals shows significant Mahalanobis distances between every pair of species (Additional file 1: Table S5 and Figure S2). The results of both tests indicate a differentiation in body morphology among the Paridae species.

**Fig. 2:**
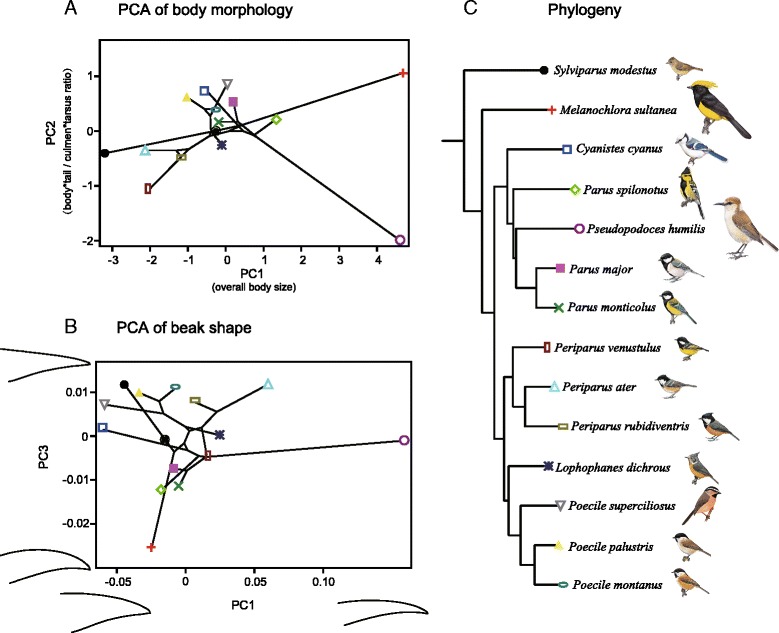
Scatters of the principal component analysis of the morphology of the 14 species examined. **a**: Scatters of the PC values for body morphology. **b**: Scatters of the PC values for beak shape. Phylogeny is projected onto the PC values in each plot. **c**: Phylogeny of the Paridae, including the 14 species examined in this study. The species illustrations refer to HBW alive: http://www.hbw.com/

The PCA performed on species mean values of linear characters shows that most of the variation is concentrated in the first two axes (Additional file 1: Table S6). PC1 explains 84.13 % of the total variance and is interpreted as the enlargement of all six characters. PC2 explains 11.06 % of the total variance and is interpreted mainly as changes in the ratio of the body and tail length to the culmen and tarsus length (Fig. 2a). Hence, PC1 can be considered an indicator of overall body size, and PC2 represents differences in the arrangement of body parts. The first two PC axes separate *Ps. humilis* (a large-bodied tit with a long tarsus and culmen) and *M. sultanea* (a large-bodied tit with a long body and tail) from the main scatter, but the rest of the species largely cluster together (Fig. 2a).

### Variation in beak shape

The result of the Procrustes ANOVA shows significant interspecific differences in both shape [*F*_(494, 12096)_ = 55, *P* < 0.0001] and size [*F*_(13, 292)_ = 110, *P* < 0.0001] of beak morphology. The CVA shows significance for most pairwise Mahalanobis distances (see Additional file 1: Table S7), suggesting differentiation in beak shape among the Paridae species.

**Fig. 3:**
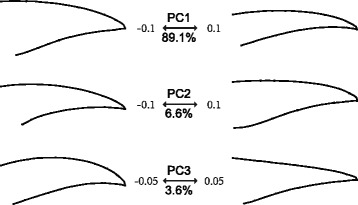
Patterns of shape change associated with PCs calculated from beak shape variation

The PCA conducted on the covariance matrix of Procrustes coordinates concentrates more than 99 % of the total variance in the first 3 axes (Fig. 3). PC1 can be interpreted as the variation between a long, slender, pointed beak shape and a short, robust, blunt shape. PC2 is associated with the relative position of the posterior-most point of the upper and lower curves of the beak, i.e., LM1 and LM2 (Fig. 1). PC3 relates to variation between beaks with straight, sharp, triangular outlines and beaks with relatively rounder and decurved outlines (Fig. 3). Because LM1 and LM2 were artificially defined rather than strictly homologous, PC2 is likely to be related to the abrasion of the horny surface on the beaks and the preserved condition of the museum specimens. In addition, this dimension of shape variation shows less interspecific difference than the other dimensions (Additional file 1: Figure S2), which makes species mean values less relevant. Therefore, the following analyses and illustrations concentrate on PC1 and PC3. These two axes separate *Ps. humilis* from the main group, whereas the remaining species form a dense and narrow cluster in the tangent shape space (Fig. 2b).

### Evolutionary changes and phylogenetic signals

The projections of the phylogenetic tree into the PC plots show some visible clusters that correspond to groups of related taxa (Fig. 2). *Sylviparus* and *Melanochlora*, as the two oldest lineages of Paridae, are connected to their sister node with long branches. On the other hand, the plots show extensive crossings of branches and some relatively long branches between related species (see *Ps. humilis*).

Significant phylogenetic signals are found in log-transformed body length and PC3 of beak shape (Additional file 1: Table S8). After correcting for overall body size, body length loses its phylogenetic signal. In addition, when *Ps. humilis*, a morphological outlier, is excluded from the analysis, body weight and size-corrected culmen length show significant phylogenetic signals.

### Covariation between morphology and altitude

**Fig. 4:**
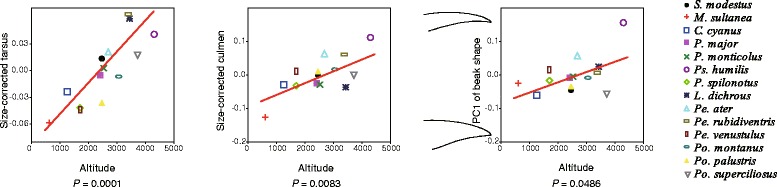
Interspecific covariation between morphological traits and altitude of the 14 species examined. Correlations of altitude with tarsus and culmen length and PC1 of beak shape are shown. Dots with different colours and shapes represent different species. Lines represent phylogenetic generalized least square (PGLS) regressions

**Fig. 5:**
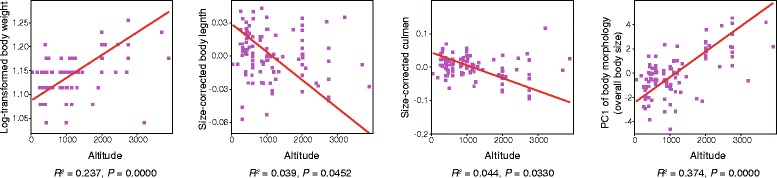
Intraspecific covariation between morphological traits and altitude within *P. major*. Correlations of altitude with body weight, body length, culmen length and PC1 of body morphology are shown. Each dot represents an individual *P. major* specimen. Lines represent ordinary least square (OLS) regressions

The results of the PGLS analysis show that across the 14 species analysed, altitude is significantly correlated with tarsus and culmen length and with the short/blunt/robust vs. long/slender/pointed dimension of beak shape variation (Fig. 4 and Additional file 1: Table S9). Within individual *P. major* specimens, altitude is correlated with multiple morphological traits (Fig. 5 and Additional file 1: Table S9). Individuals at higher elevations tend to have a greater body weight and larger overall body size, as well as a longer body and culmen (Fig. 5).

### Character divergence and distribution overlap

Mantel tests detected a significant positive correlation between the morphological distance and the distributional distance (both geographic and altitudinal) after controlling for phylogenetic relationships (Additional file 1: Table S10). The results indicate that Paridae species that overlap more in their distributional range tend to have a higher level of morphological similarity than is observed for species with greater distributional differences. This pattern remains when *Ps. humilis* is excluded from the analysis.

## Discussion

In this study we quantified morphological variation among 14 species from the main clades of Paridae using both traditional and geometric morphometrics. We also assessed the contribution of phylogenetic relatedness, altitudinal distribution and/or species interactions to morphological evolution. Two major aspects of variation in body morphology were identified: overall body size and body shape (the ratio of body and tail length to tarsus and culmen length). Beak shape variation was concentrated on the long/slender/pointed vs. short/robust/blunt axis, which is in agreement with previous studies on the beak morphology of Darwin’s finches [30, 64]. In addition, we extracted other dimensions of shape change, such as the curvature of the upper mandible. Both body morphology and beak shape identify *Ps. humilis* and *M. sultanea* as obvious outliers, which can be visualized in the shape space (Fig. 2). Typical “tit-like” parids are known to be outwardly uniform in shape [16, 19], but our quantitative methods separated most of the species based on body morphology (Additional file 1: Figure S2A). However, most species do overlap in the beak variation shape space (Additional file 1: Figure S2C). Beak shape appears to vary mostly along the long/slender/pointed vs. short/robust/blunt axis, which discriminates *Ps. humilis* from the main cluster (Fig. 2). Paridae species are relatively conservative in beak shape, which is in contrast to other adaptive radiations that harbour a greater diversity of beak morphology, such as Darwin’s finches [13], Malagasy vangas [10] and Hawaiian honeycreepers [14].

Only body length and the curvature of the beak show a significant phylogenetic signal. The signal in body length is lost after size correction, which indicates that the signal associated with body length is primarily due to overall body size. Regarding beak morphology, the curvatures of beak outline are more phylogenetically conserved than the slenderness of the beak. The exclusion of *Ps. humilis* increases the phylogenetic signal in the morphological characters, such as body size and beak morphology, which suggests that the phylogenetic signals in morphological traits may be interfered by the discrepancy between the striking morphological divergence and close phylogenetic relationships. The morphological difference between *Ps. humilis* and its closest relatives (*P. major* and *P. monticolus*) is larger than that among other genera of Paridae [21].

Altitude is significantly correlated with tarsus length and beak morphology across the 14 species in this study. In addition, there is an intraspecific body-size cline along the altitudinal gradient within *P. major,* a species that covers a wide altitudinal range. Both results suggest that morphological traits of Paridae covary with altitudinal distributions. Altitude relates to the range of habitat occupied [65, 66] and acts as a selective power on morphology. The long/slender/pointed and short/blunt/robust beak morphs reflect foraging behaviours of probing on ground and pecking in wooded habitats, respectively [8]. In addition, variation in tarsus length, which was also discovered within core Corvoidea and *Phylloscopus* warblers [65, 66], is also considered to reflect adaptations to foraging in more open habitats at higher elevations, especially hopping and walking on the ground. The intraspecific size cline may be a response to the temperature variation that occurs along altitudinal gradients [67, 68].

Both geographical separation and ecological differences between populations contribute to speciation in birds [69]. Here, we speculate that altitude is an ecological factor that provides different selective pressures and promote the divergence of Paridae. *Pseudopodoces humilis*, an extremely morphologically deviant species within Paridae, is an example of speciation promoted by altitude and related habitats. The uplift of the QTP created the high steppes habitat and provided an “open” niche, which triggered the morphological evolution of the ancestor of *Ps. humilis* [21, 35]. Rather than being similar to its forest-living parid relatives, it is instead morphologically convergent to ground jays [21] and mynas, which are also predominantly open-country ground foragers [35]. The deviant appearance of *Ps. humilis* reflects adaptations to a novel adaptive zone [21, 35], including a larger body size for preserving heat; increased tarsal length for terrestrial locomotion; and a long, slender, decurved beak for probing, pecking and digging vigorously in soft earth, turf, yak dung and decaying animal corpses to find small invertebrates and larvae [19, 20]. The different selective pressures of the high steppes and the forests drive the divergence between *Ps. humilis* and other parids.

Closely related sympatric species usually need to diverge in ecological niches to avoid resource competition [24, 70, 71]. Previous studies have discovered niche divergence in sympatric Paridae species [72, 73] and character displacement in some local communities [39, 74]. This is supported by our results which discovered significant differences for body morphology and/or beak shape among species (Additional file 1: Table S5 and S7), albeit their superficial similarity. On the other hand, the results of partial Mantel test found further morphological divergence with distributional differences at a larger scale (Additional file 1: Table S10). Paridae shows patterns of morphological divergence related to differences in distribution ranges, likely driven by adaptation to different habitats and selective pressures at different ranges.

## Conclusions

In this study, we quantified the morphological variation of 14 Paridae species distributed in China. The basic features for discrimination are overall body size, the ratio of body and tail length to culmen and tarsus length, and beak shape (long/slender/pointy vs. short/robust/blunt). These dimensions identify *Ps. humilis* and *M. sultanea* as obvious outliers. Body morphology shows extensive variation and can separate most species, while beak shape has evolved mostly along the long/slender/pointy vs. short/robust/blunt dimension. Only body length and beak curvature show a phylogenetic signal. The low level of phylogenetic signal in the morphological data may be due to the close phylogenetic relationship and considerable morphological divergence between *Ps. humilis* and its closest relatives. Altitude is correlated with multiple traits both across and within species. We speculate that the altitudinal gradient is an important factor promoting morphological divergence. The deviant appearance of *Ps. humilis*, including its large body size, long tarsus and long, slender, decurved beak, reflects its adaptation to the high-altitude steppes created by the uplift of the Qinghai-Tibet Plateau. We also show here that Paridae species with greater distributional difference tend to have a higher level of morphological divergence, which is probably due to different selective pressures provided by habitats at different ranges.

## Additional files

Additional file 1: Supplementary materials. Included are 10 supplementary tables (Table S1-S10) and 2 supplementary figures (Figures S1-S2). (PDF 2118 kb)

Additional file 2: Body measurements. Data for the linear measurements of all specimens involved in this study are shown, including body weight, body length, wing length, tail length, tarsus length and culmen. The altitude for each specimen is also included. (CSV 27 kb)

Additional file 3: Coordinates of landmarks for geometric morphometric analysis of beak shape. (TXT 261 kb)

Additional file 4: Phylogeny of the 14 Paridae species analysed in this study. (TRE 525 bytes)
