# Supplementary material for: Evolution of body morphology and beak shape revealed by a morphometric analysis of 14 Paridae species
Source: Front Zool. 2016 Jun 29;13:30. doi: 10.1186/s12983-016-0162-0 (PMC4928266; doi:10.1186/s12983-016-0162-0)
Supplement: Supplementary file 1 — Supplementary materials. Included are 10 supplementary tables (Table S1-S10) and 2 supplementary figures (Figures S1-S2). (PDF 2118 kb) [file 12983_2016_162_MOESM1_ESM.pdf]

Additional File 1. Supplementary materials.

Table S1. List of Paridae species in this study.

Table S2. Methods for the linear morphological measurements.

Table S3. List of sequences and GenBank accession numbers used in the phylogenetic reconstruction.

Table S4. Summary statistics for body morphology of Paridae species examined.

Table S5 Mahalanobis distances for body morphology among species.

Table S6. Results of the PCA of body morphology.

Table S7. Mahalanobis distances for beak shape among species.

Table S8. Phylogenetic signals for each morphological character.

Table S9. Covariation between morphology and altitude.

Table S10. Results of the Mantel test for the correlation between character divergence and distributional distances.

Figure S1. Box plots of body characters of the 14 Paridae species examined.

Figure S2. Results of the canonical variate analysis (CVA) on the morphology of individuals from the 14 species examined. The CVA was able to identify axes that concentrated most of the interspecific variation. A: Plot of CV1 and CV2 for body morphology. B: Loading coefficients of CV1 and CV2. CV1 explains 77.92 % of the total variance and is interpreted as the ratio of body weight, tarsus and culmen length to tail length. CV2 explains 13.72% of the total variance and is interpreted as the wing/tail length ratio. C. Plot of CV1 and CV2 for beak shape. CV1 explains 53.07 % of the total variance and is associated with the variation from a long, slender, pointed beak to a short, blunt, robust beak. CV2 explains 15.27 % of the total variance and is associated with beak shape variation from a straight outline to a rounded outline. CV3 explains 12.92 % of the total variance and is associated with the curvature of the beak outline near the tip, as well as relative position of landmarks 1 and 2. This axis of shape variation shows the least interspecific difference.

Table S1. List of Paridae species in this study.

Common name	Scientific name	Sample size	
		Body morphology	Beak shape
Yellow-browed Tit	*Sylviparus modestus*	7	7
Sultan Tit	*Melanochlora sultanea*	8	9
Azure Tit	*Cyanistes cyanus*	11	11
Great Tit	*Parus major*	104	69
Green-backed Tit	*Parus monticolus*	46	29
Ground Tit	*Pseudopodoces humilis*	41	27
Yellow-cheeked Tit	*Parus spilonotus*	16	18
Grey-crest Tit	*Lophophanes dichrous*	6	8
Coal Tit	*Periparus ater*	11	11
Rufous-vented Tit	*Periparus rubidiventris*	22	22
Yellow-bellied Tit	*Periparus venustulus*	30	33
Willow Tit	*Poecile montanus*	18	19
Marsh Tit	*Poecile palustris*	49	38
White-browed Tit	*Poecile superciliosus*	7	5
Total		376	306

Table S2. Methods for the linear morphological measurements.

Character	Method
Body weight	Weighed in the field by collectors.
Body length	Measured in the field by collectors.
Wing length	The distance between the bend of wing to the tip of longest primary, measured on the closed wing.
Tail length	The distance from the root of central pairs of tail-feathers to tip of longest tail-feather, measured when the tail is naturally folded.
Tarsus length	The distance from the notch on the back of the inter-tarsal joint to the lower edge of the last complete scale before the toes diverge.
Culmen	The distance from the tip of the bill to the distal edge of the nostrils.

References:

Svenson L, Identification guide to European Passerines; London; British Trust for Ornithology; 1992.

Table S3. List of sequences and GenBank accession numbers used in the phylogenetic reconstruction.

Scientific name	*Myo*	Ref	*ODC*	Ref	*ND2*	Ref	*cytb*	Ref
Sylviparus modestus	KF183651	[1]	KF183738	[1]	KF183827	[1]	HM185371	[2]
Melanochlora sultanea	KF183652	[1]	KF183739	[1]	KF183828	[1]	AY308721	[3]
Cyanistes cyanus	KF183656	[1]	KF183743	[1]	KF183832	[1]	HM185352	[2]
Parus major	AY228310	[4]	KF183747	[1]	AY136587	[5]	HM185345	[2]
Parus monticolus	KF183658	[1]	KF183746	[1]	KF183835	[1]	HM185349	[2]
Pseudopodoces humilis	KF183657	[1]	KF183744	[1]	KF183833	[1]	HM185372	[2]
Parus spilonotus	KF183664	[1]	KF183752	[1]	KF183840	[1]	HM185351	[2]
Lophophanes dichrous	KF183704	[1]	KF183792	[1]	KF183880	[1]	HM185367	[2]
Periparus ater	KF183698	[1]	KF183786	[1]	KF183874	[1]	HM185355	[2]
Periparus rubidiventris	KF183701	[1]	KF183789	[1]	KF183877	[1]	AY308725	[3]
Periparus venustulus	KF183697	[1]	KF183785	[1]	KF183873	[1]	HM185353	[2]
Poecile montanus	KF183721	[1]	KF183809	[1]	KF183897	[1]	HM185363	[2]
Poecile palustris	KF183719	[1]	KF183807	[1]	KF183895	[1]	HM185358	[2]
Poecile superciliosus	KF183714	[1]	KF183802	[1]	KF183890	[1]	AF347952	[3]
Regulus regulus	DQ466835	[6]	EU680761	[7]	KF183821	[1]	AJ004762	[7]

References:

1. Johansson US, Ekman J, Bowie RCK, Halvarsson P, Ohlson JI, Price TD et al. A complete multilocus species phylogeny of the tits and chickadees (Aves: Paridae). Mol Phylogenet Evol. 2013;69(3):852-60.

2. Dai CY, Chen K, Zhang RY, Yang XJ, Yin ZH, Tian HJ et al. Molecular phylogenetic analysis among species of Paridae, Remizidae and Aegithalos based on mtDNA sequences of *COI* and *cyt b*. Chinese Birds. 2010;1(2):112-23.

3. Gill FB, Slikas B, Sheldon FH. Phylogeny of titmice (Paridae): II. Species relationships based on sequences of the mitochondrial cytochrome-B gene. Auk. 2005;122(3):121-43.

4. Ericson PGP, Johansson US. Phylogeny of Passerida (Aves : Passeriformes) based on nuclear and mitochondrial sequence data. Mol Phylogenet Evol. 2003;29(1):126-38.

5. Sefc KM, Payne RB, Sorenson MD. Phylogenetic relationships of African sunbird-like warblers:

Moho (*Hypergerus atriceps*), Green Hylia (*Hylia prasina*) and Tit-hylia (*Pholidornis rushiae*). Ostrich. 2003;74(1-2):8-17.

6. Johansson US, Fjeldsa J, Bowie RCK. Phylogenetic relationships within Passerida (Aves : Passeriformes): a review and a new molecular phylogeny based on three nuclear intron markers. Mol Phylogenet Evol. 2008;48(3):858-76.
7. Zuccon D, Cibois A, Pasquet E, Ericson PGP. Nuclear and mitochondrial sequence data reveal the major lineages of starlings, mynas and related taxa. Mol Phylogenet Evol. 2006;41(2):333-44.

Table S4. Summary statistics for body morphology of Paridae species examined.

	Sample size	Body weight/g	Body length mm	Wing length mm	Tail length mm	Tarsus length mm	Culmen length mm
Sylviparus modestus	7	7.20±0.15	88.12±1.23	55.90±1.34	37.97±0.76	14.23±0.22	5.71±0.05
Melanochlora sultanea	8	39.13±1.16	196.38±2.09	106.36±1.49	92.74±1.50	21.55±0.27	11.10±0.25
Cyanistes cyanus	11	12.09±0.21	123.36±2.12	65.95±0.49	59.17±1.21	15.61±0.19	7.15±0.13
Parus major	104	13.92±0.13	131.65±0.68	68.49±0.39	62.40±0.41	17.20±0.09	7.81±0.05
Parus monticolus	46	13.35±0.24	123.09±1.25	66.58±0.36	55.04±0.46	17.15±0.08	7.56±0.06
Pseudopodoces humilis	41	39.51±0.57	162.73±1.58	88.47±0.39	60.86±0.62	27.16±0.12	19.31±0.30
Parus spilonotus	16	19.19±0.31	137.75±2.22	79.02±0.65	60.68±0.77	17.54±0.18	9.21±0.11
Lophophanes dichrous	6	12.67±0.56	114.83±3.07	71.20±1.44	51.78±1.29	19.15±0.38	7.22±0.24
Periparus ater	11	8.18±0.35	100.00±1.39	58.17±0.60	43.69±1.01	15.13±0.28	7.10±0.19
Periparus rubidiventris	22	8.91±0.25	103.86±1.20	64.78±0.55	46.74±0.45	17.17±0.13	7.40±0.10
Periparus venustulus	30	11.07±0.17	97.17±0.75	63.85±0.34	35.01±0.42	14.46±0.08	7.47±0.06
Poecile montanus	18	12.11±0.31	120.56±1.41	64.02±0.44	59.27±0.63	16.24±0.14	7.95±0.11
Poecile palustris	49	10.59±0.16	118.98±1.49	62.55±0.40	55.58±0.86	14.50±0.09	7.28±0.06
Poecile superciliosus	7	11.86±0.63	129.86±2.44	64.39±0.65	66.54±1.13	17.03±0.19	7.54±0.08
Total	376	15.85±0.48	126.54±1.09	69.20±0.51	56.54±0.53	17.57±0.18	8.90±0.19
$F_{(13,376)}$		586.972	203.478	231.813	153.200	454.908	670.677
P-value		< 0.0001	< 0.0001	< 0.0001	< 0.0001	< 0.0001	< 0.0001

The value of each characteristic is given as mean ± SE.

Table S5 Mahalanobis distances for body morphology among species.

	Sy. modestus	*Me. sultanea*	*Cy. cyanus*	*P. major*	*P. monticolus*	*Ps. humilis*	*P. spilonotus*	*Lo. dichrous*	*Pe. ater*	*Pe. rubidiventris*	*Pe. venustulus*	*Po. montanus*	*Po. palustris*
Me. sultanea	412.7*												
Cy. cyanus	56.52*	198.31*											
P. major	86.60*	149.37*	6.93*										
P. monticolus	64.97*	167.42*	8.61*	3.50*									
Ps. humilis	630.85*	188.41*	434.68*	345.83*	343.95*								
P. spilonotus	141.54*	78.66*	41.16*	25.43*	25.74*	241.79*							
Lo. dichrous	72.95*	176.45*	38.33*	27.66*	17.07*	348.23*	44.31*						
Pe. ater	15.17*	322.08*	27.03*	42.99*	29.97*	490.56*	87.93*	49.35*					
Pe. rubidiventris	32.78*	262.03*	31.33*	34.97*	22.35*	423.50*	68.27*	17.80*	12.69*				
Pe. venustulus	48.49*	297.03*	74.03*	89.70*	63.35*	432.60*	78.27*	75.34*	41.98*	52.36*			
Po. montanus	69.02*	197.24*	5.32*	5.37*	7.81*	380.33*	40.18*	40.76*	27.20*	31.00*	81.81*		
Po. palustris	46.97*	246.66*	5.13*	19.79*	20.09*	474.91*	58.63*	60.76*	18.62*	36.05*	65.88*	9.37*	
Po. superciliosus	89.74*	200.75*	11.16*	7.69*	15.86*	412.23*	58.85*	46.27*	45.15*	42.90*	126.41*	6.77*	20.93*

"*" indicates statistical significance ($P < 0.05$). P-values were processed with Bonferroni-Holm correction to minimize type I error.

Table S6. Results of the PCA of body morphology.

	Eigenvalue	% variance	Loading coefficients of morphological characteristics					
			Body weight	Body length	Wing length	Tail length	Tarsus length	Culmen length
PC1	5.0479	84.13	0.44	0.43	0.42	0.36	0.40	0.39
PC2	0.6636	11.06	-0.10	0.31	0.03	0.70	-0.39	-0.51
PC3	0.1632	2.72	-0.34	0.01	-0.66	0.41	0.48	0.23

Table S7. Mahalanobis distances for beak shape among species.

	Sy. modestus	*Me. sultanea*	*Cy. cyanus*	*P. major*	*P. monticolus*	*Ps. humilis*	*P. spilonotus*	*Lo. dichrous*	*Pe. ater*	*Pe. rubidiventris*	*Pe. venustulus*	*Po. montanus*	*Po. palustris*
Me. sultanea	8.20*												
Cy. cyanus	5.03*	6.47*											
P. major	4.83*	5.66*	3.86*										
P. monticolus	5.02*	5.70*	4.22*	1.68*									
Ps. humilis	10.79*	9.91*	10.38*	8.87*	8.86*								
P. spilonotus	5.11*	6.04*	4.01*	1.96*	2.23*	9.33*							
Lo. dichrous	5.17*	6.50*	4.96*	2.57*	2.66*	8.44*	3.64*						
Pe. ater	6.11*	8.29*	6.62*	4.75*	4.96*	7.86*	5.30*	4.10*					
Pe. rubidiventris	4.54*	7.28*	4.52*	2.82*	3.17*	8.91*	3.29*	2.79*	3.96*				
Pe. venustulus	4.86*	7.58*	5.75*	4.09*	4.03*	9.34*	4.16*	4.25*	4.17*	4.35*			
Po. montanus	4.03*	7.46*	4.21*	3.05*	3.45*	9.04*	3.56*	3.23*	4.01*	2.43*	4.34*		
Po. palustris	4.42*	7.44*	3.76*	3.37*	4.01*	10.32*	3.67*	4.01*	5.62*	3.11*	5.23*	2.55*	
Po. superciliosus	4.28*	7.11*	2.69	4.01*	4.29*	10.57*	4.50*	4.75*	6.43*	4.27*	5.96*	3.50*	3.40

"*" indicates statistical significance ($P < 0.05$). P-values were processed with Bonferroni-Holm correction to minimize type I error.

Table S8. Phylogenetic signals for each morphological character.

Character	14 species				13 species (Without *Ps. humilis*)			
	K	*P*-value	λ	*P*-value	K	*P*-value	λ	*P*-value
Log10(body weight)	1.0915	0.065	0.9999	0.063	1.1022	0.088	0.9999	0.046*
Log10(Body length)	1.1182	0.036	0.9999	0.037*	1.0925	0.086	0.9999	0.045*
Log10(Wing length)	1.0423	0.094	0.9999	0.067	1.0496	0.109	0.9999	0.070
Log10(Tail length)	0.9580	0.095	0.9999	0.102	0.9547	0.109	0.9999	0.128
Log10(Tarsus length)	0.8754	0.174	0.0001	1.000	0.7636	0.320	0.0001	1.000
Log10(Culmen length)	1.0133	0.130	0.9999	0.395	1.0718	0.087	0.9999	0.072
Size-corrected body length	0.7511	0.333	0.0001	1.000	0.7602	0.327	0.0001	1.000
Size-corrected wing length	0.9004	0.171	0.0001	1.000	0.7972	0.231	0.0001	1.000
Size-corrected tail length	0.6799	0.509	0.0001	1.000	0.6962	0.407	0.0001	1.000
Size-corrected tarsus length	0.7372	0.370	0.0001	1.000	0.6891	0.477	0.0001	1.000
Size-corrected culmen length	1.0585	0.069	0.9999	0.246	1.2689	0.038	0.9999	0.038*
PC1 of body morphology	1.0623	0.056	0.9999	0.075	1.0515	0.104	0.9999	0.059
PC2 of body morphology	0.8347	0.196	0.0001	1.000	0.8851	0.153	0.9999	0.260
PC1 of beak shape	0.8979	0.155	0.0001	1.000	0.8556	0.188	0.0001	1.000
PC3 of beak shape	1.1044	0.059	0.9999	0.040*	1.1407	0.046	0.9999	0.044*

P-value is result for the null hypothesis of no phylogenetic signal. “*” indicates statistical significance ($P < 0.05$).

Table S9. Covariation between morphology and altitude.

Character	PGLS of species means	Individuals within *P. major*	
	P-value	R^2	*P*-value
Log10(body weight)	0.7637	0.237	0.0000*
Size-corrected body length	0.6004	0.039	0.0452*
Size-corrected wing length	0.2302	0.034	0.0596
Size-corrected tai length	0.7505	0.004	0.5099
Size-corrected tarsus length	0.0001*	0.004	0.5250
Size-corrected culmen length	0.0083*	0.044	0.0330*
PC1 of body morphology	0.4941	0.374	0.0000*
PC2 of body morphology	0.0803	0.000	0.9504
PC1 of beak shape	0.0486*	0.000	0.5874
PC3 of beak shape	0.0783	0.003	0.0576

"*" indicates statistical significance ($P < 0.05$).

Table S10. Results of the Mantel test for the correlation between character divergence and distributional distances.

Dataset	Distribution	Body morphology		Beak shape	
		R	*P*-value	*R*	*P*-value
14 species	Geographic	0.235	0.014 *	0.428	0.019 *
	Altitudinal	0.253	0.011 *	0.457	0.015 *
13 species	Geographic	0.272	0.005 *	0.466	0.013 *
(Without *Ps.humilis*)	Altitudinal	0.246	0.017 *	0.399	0.029 *

"*" indicates statistical significance ($P < 0.05$).

Figure S1

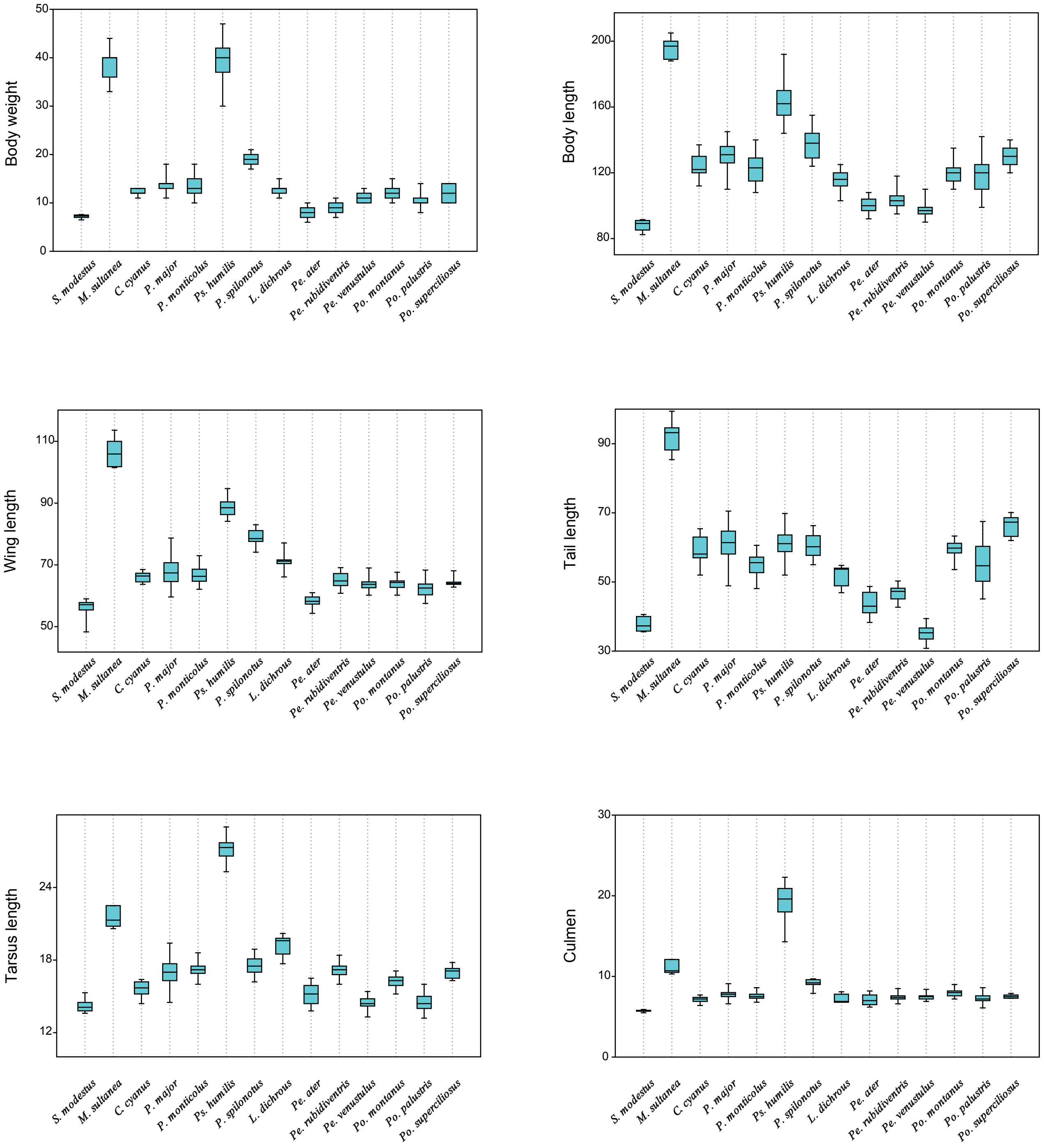

Figure S1. Box plots of body characters of the 14 Paridae species examined.

Figure S2

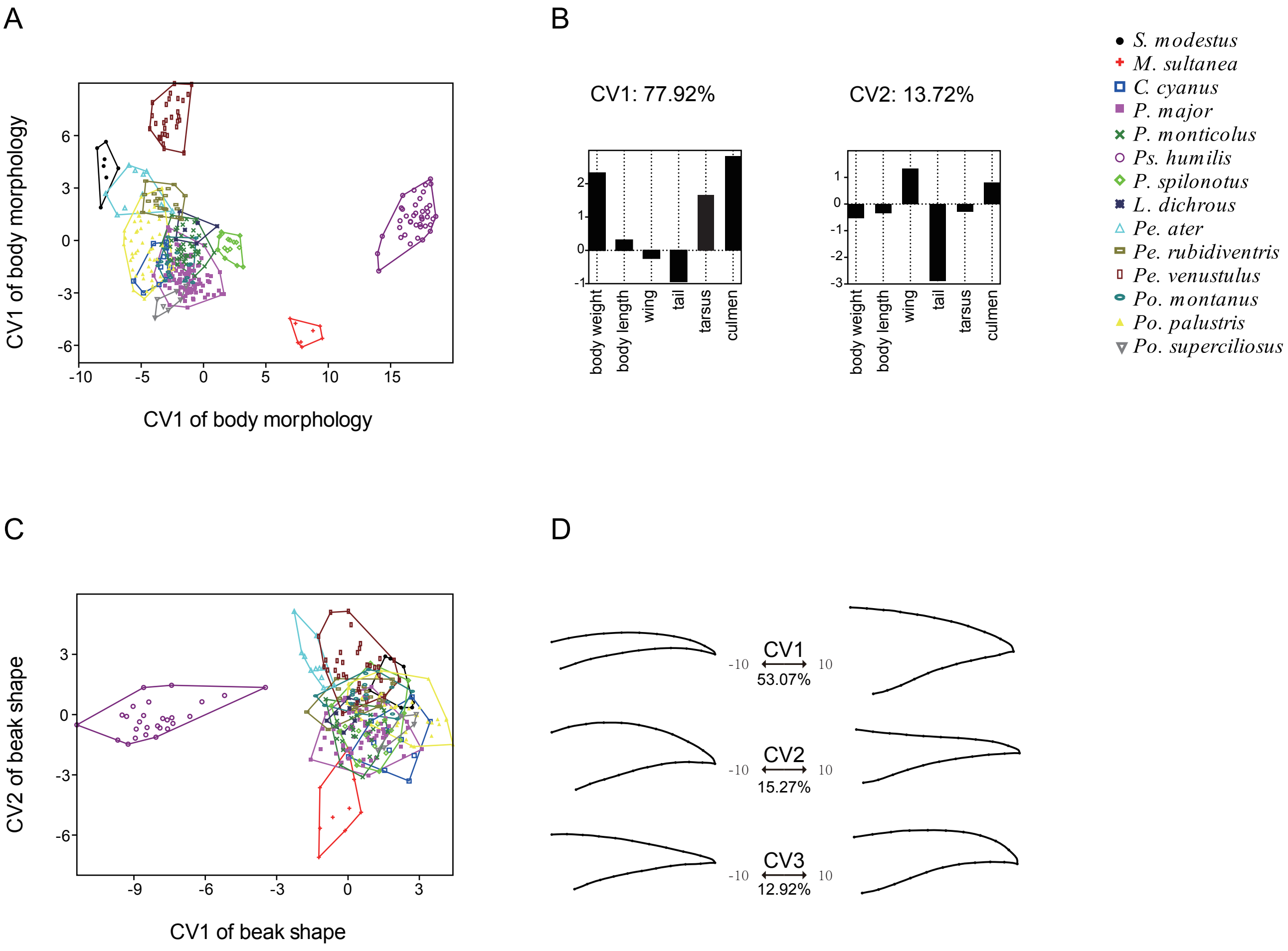

Figure S2. Results of the canonical variate analysis (CVA) on the morphology of individuals from the 14 species examined. The CVA was able to identify axes that concentrated most of the interspecific variation. A: Plot of CV1 and CV2 for body morphology. B: Loading coefficients of CV1 and CV2. CV1 explains 77.92 % of the total variance and is interpreted as the ratio of body weight, tarsus and culmen length to tail length. CV2 explains 13.72% of the total variance and is interpreted as the wing/tail length ratio. C. Plot of CV1 and CV2 for beak shape. CV1 explains 53.07 % of the total variance and is associated with the variation from a long, slender, pointed beak to a short, blunt, robust beak. CV2 explains 15.27 % of the total variance and is associated with beak shape variation from a straight outline to a rounded outline. CV3 explains 12.92% of the total variance and is associated with the curvature of the beak outline near the tip, as well as relative position of landmarks 1 and 2. This axis of shape variation shows the least interspecific difference.
